# Anti-Cancer Activity of *Cannabis sativa* Phytocannabinoids: Molecular Mechanisms and Potential in the Fight against Ovarian Cancer and Stem Cells †

**DOI:** 10.3390/cancers14174299

**Published:** 2022-09-01

**Authors:** Hinanit Koltai, Nurit Shalev

**Affiliations:** 1 Institute of Plant Science, Agriculture Research Organization, Volcani Institute, Rishon LeZion 7505101, Israel; 2 The Mina and Everard Goodman Faculty of Life Sciences, Bar-Ilan University, Ramat Gan 5290002, Israel

**Keywords:** ovarian cancer, stem cells, ovarian cancer stem cells, cannabis, phytocannabinoids, signaling pathways, resistance mechanisms, therapeutic properties

## Abstract

**Simple Summary:**

Ovarian cancer is the most lethal gynecological malignancy. Cancer stem cells have been implicated in tumor initiation, progression, and invasion, as well as tumor recurrence, metastasis, and drug resistance. Cannabis is used worldwide to alleviate numerous symptoms associated with various medical conditions. Phytocannabinoids, produced by cannabis, were shown to have anti-cancer activity in cell lines and animal models, but also the potential to increase other drugs’ adverse effects. Yet, very few studies have examined the effectiveness of cannabis compounds against ovarian cancer. Cannabis compounds have been shown to affect genetic pathways and biological processes related to development of ovarian cancer stem cells. Phytocannabinoid-based treatments might be used to disrupt cancer stem cell homeostasis and thereby to prevent chemotherapy resistance. The potential benefits of the combination of chemotherapy with phytocannabinoid treatment could be examined in ovarian cancer patients.

**Abstract:**

Ovarian cancer (OC) is the most lethal gynecological malignancy, with about 70% of cases diagnosed only at an advanced stage. *Cannabis sativa*, which produces more than 150 phytocannabinoids, is used worldwide to alleviate numerous symptoms associated with various medical conditions. Recently, studies across a range of cancer types have demonstrated that the phytocannabinoids Δ^9^-trans-tetrahydrocannabinol (THC) and cannabidiol (CBD) have anti-cancer activity in vitro and in vivo, but also the potential to increase other drugs’ adverse effects. THC and CBD act via several different biological and signaling pathways, including receptor-dependent and receptor-independent pathways. However, very few studies have examined the effectiveness of cannabis compounds against OC. Moreover, little is known about the effectiveness of cannabis compounds against cancer stem cells (CSCs) in general and OC stem cells (OCSCs) in particular. CSCs have been implicated in tumor initiation, progression, and invasion, as well as tumor recurrence, metastasis, and drug resistance. Several hallmarks and concepts describe CSCs. OCSCs, too, are characterized by several markers and specific drug-resistance mechanisms. While there is no peer-reviewed information regarding the effect of cannabis and cannabis compounds on OCSC viability or development, cannabis compounds have been shown to affect genetic pathways and biological processes related to CSCs and OCSCs. Based on evidence from other cancer-type studies, the use of phytocannabinoid-based treatments to disrupt CSC homeostasis is suggested as a potential intervention to prevent chemotherapy resistance. The potential benefits of the combination of chemotherapy with phytocannabinoid treatment should be examined in ovarian cancer patients.

## 1. Introduction

Ovarian cancer (OC) is the most lethal and the second most common gynecological malignancy in the western world. Around 70% of OC cases are only diagnosed at an advanced stage, and late-stage OC is incurable in most cases [1]. OC is the fifth leading cause of cancer-related deaths among women and the seventh most common cancer globally (15–20 cases per 100,000) [2]. Initially, epithelial OC is associated with subtle symptoms, including abdominal pain and distension. OC typically presents in postmenopausal women, and many women go at least six months before being diagnosed. No routine screening test has been found to be effective for OC, and mortality is not reduced by population-level monitoring [3]. The standard of care for the advanced disease remains surgery and platinum-based cytotoxic chemotherapy [4]. Additional treatments include targeted therapies that disrupt cancer-related processes necessary for tumor growth, division, and spread. These include, for example, the use of neovasculature and angiogenesis inhibitors, both processes of tumor growth and progression; the use of Poly (ADP-ribose) polymerase (PARP) inhibitors that block DNA damage repair; inhibitors of inhibitors (e.g., tropomyosin receptor kinase (TRK)) of survival pathway activators (e.g., MAPK/ERK and PI3K/AKT pathways); and hormone therapy [5,6]. Nevertheless, disease relapse is expected in about 80% of cases, on average, after 24 months. Eventually, multidrug resistance develops, and very few women survive five years after diagnosis.

## 2. Cannabis Compounds

*Cannabis sativa* is used worldwide to lessen various symptoms accompanying medical conditions [7]. In each *C. sativa* strain, several dozen compounds are produced, and in total, around 600 different molecules are biosynthesized in the species, including more than 150 phytocannabinoids and hundreds of terpenes and flavonoids [8,9,10]. Several phytocannabinoids have anti-cancer activity in vitro and in vivo, including in skin, prostate, lung, breast, and glioma cancer cells [11,12,13]. Phytocannabinoids act in many ways against cancers, e.g., by inhibiting cell proliferation and migration, inhibiting angiogenesis, and inducing apoptosis [11,12,13]. However, to exploit the full potential of cannabis, the active molecules should be defined and the cellular and molecular mechanisms that underlie cannabis’s anti-cancer activity should be better understood (see Appendix A for methodology).

Cannabis produces a large number of compounds, including more than 150 phytocannabinoids, a vast array of terpenes, and cannflavins, which are geranylated (C10) and prenylated (C5) flavones [8,9,10,14]. Phytocannabinoids derive from meroterpenoids with a resorcinyl core structure and are aromatic oxygenated hydrocarbons. They bear either isoprenyl, alkyl, or aralkyl substitutions. The alkyl side chain usually contains an odd number of carbon atoms [8,9]. Phytocannabinoids are produced in the plant in their acid form [9]. Cannabigerolic acid (CBGA; Table 1) is the core intermediate that diverges to form the phytocannabinolic acids [8,15]. Phytocannabinoids may be decarboxylated to the active form, most commonly by heat treatment. Among the decarboxylated phytocannabinoids, cannabidiol (CBD; Table 1) and the primary psychoactive molecule Δ^9^-trans-tetrahydrocannabinol (THC; Table 1) are the most abundant [8,15]. Cannabichromenic acid (CBCA; Table 1), which decarboxylases to cannabichromene (CBC; Table 1), is another CBGA-derived phytocannabinolic acid; CBC is usually less abundant than THC or CBD in cannabis products [8,9,10].

## 3. The Endocannabinoid System and Cancer

The endocannabinoid system (ECS) is widely distributed in the body. It serves as a signaling network that is important in maintaining homeostatic balance. It regulates various physiological processes, including immunomodulation and synaptic transmission [16]. The ECS consists of endocannabinoids that are endogenous ligands to cannabinoid receptors (receptors are detailed below) and metabolic enzymes [16]. Various diseases are associated with dysregulation of the ECS, including diabetes, obesity, depression and anxiety, neurodegenerative disorders, inflammation, schizophrenia, multiple sclerosis, cardiovascular diseases, glaucoma, and cancer [17,18]. In numerous types of cancers, ECS activity is altered [18,19,20]. It has been suggested that targeting the ECS could lead to new approaches to the treatment of various pathological conditions, including cancer [18,21,22].

## 4. Cannabinoid Receptors and Their Activation

Activation of the ECS partially depends on the binding of endo-, phyto- or synthetic cannabinoids to the cannabinoid receptors, members of the seven-transmembrane G-protein coupled receptor (GPCR) superfamily. Two types of cannabinoid receptors are recognized and abundant in the body: cannabinoid receptor types 1 and 2 (CB1 and CB2, respectively) [23]. The CB1 receptor is highly expressed in the brain, including in the hippocampus, basal ganglia nuclei, cortex, and cerebellum. These receptors are mainly localized to neuron terminals, where they mediate the inhibition of the release of neurotransmitters. The CB1 receptor is also expressed in other cell types and organs, but to a lesser extent [13]. The CB2 receptor is abundantly expressed in several organs, including the lung and testes, and in immune system organs and cells including the spleen, thymus, tonsils, macrophages, and leukocytes. Its presence in the adult brain is somewhat controversial [13].

Other phytocannabinoid receptors include GCPRs and ion channels such as G-protein-coupled receptor 55 (GPR55), the TRP ankyrin (TRPA) family, the transient receptor potential vanilloid (TRPV) family, and peroxisome proliferator-activated receptors (PPARs) [24,25]. 

THC binds to both CB1 and CB2 receptors as an agonist (activator). CB1 activation by THC is associated with catalepsy, hypothermia, desensitization of pain, the suppression of locomotor activity, and appetite enhancement. CB2 activation by THC is thought to have pain relief and anti-inflammatory effects [26]. CBD binds CB1 as an antagonist and may counteract THC activity. This counter activity is suggested to reduce the unwanted side effects associated with THC treatment, including intoxication, tachycardia, anxiety, and sedation. CBD also acts as an agonist for TRPV1 and 5-HT1A receptors, leading to anti-inflammatory, anti-psychotic, and anti-convulsive effects [26].

## 5. Cannabinoids Anti-Cancer Activity

Phytocannabinoids have been demonstrated by multiple studies to have potential anti-cancer properties. They inhibit cell migration and proliferation, induce cell death, reduce angiogenesis, and inhibit the invasiveness of cancer cells of, e.g., the skin, breast, lung, prostate, and brain [19,20,27]. Phytocannabinoids trigger cancer cell death via several signal transduction pathways, including cell cycle arrest, ER stress, oxidative stress, autophagy, and apoptosis [19,20,27].

More specifically, evidence has been accumulated regarding the anti-cancer activity of the most abundant phytocannabinoids of *C. sativa*: THC and CBD, and related synthetic compounds (e.g., the CB1/CB2-mixed agonists HU-210 and WIN-55 212-2). THC induces apoptosis in different cancer cell types in vitro and in vivo and inhibits angiogenesis and the growth of some tumors [20,27,28,29,30]. For example, through the activation of CB1 and CB2 receptors, THC reduces cell survival and proliferation and induces apoptosis of glioblastoma multiforme (GBM) cells in vitro [31]. It was also shown to inhibit the GBM cell-based xenograft growth in mice and rats in vivo [13,32]. 

CBD inhibits cancer cell proliferation, induces apoptosis, and inhibits cell invasion, metastasis, and angiogenesis in many cancer types in vitro and in vivo [13,19]. For example, CBD inhibits the invasiveness of breast cancer cells [33] and reduces the growth of GBM tumors [34]. However, in most cases, CBD does not interact with CB1 and CB2 receptors with high affinity. Thus, the initial target site (s) of CBD anti-cancer activity is/are not well-defined [13]. CBC was demonstrated to be potent against breast and prostate cancer cells [35] and act synergistically with THC against bladder cancer cells [36]. 

It should be noted, however, that a considerable fraction of information on the effects of phytocannabinoids has been obtained from studies in cancer cell cultures in which THC and CBD are directly added to culture media. Therefore, pharmacological information and metabolism of these compounds should be considered for any phytocannabinoids-based anti-cancer treatment. 

THC is metabolized in the liver by cytochrome P450 (CYP450) enzymes to the active metabolites 11-hydroxy-THC (11-OH-THC) and THC-COOH; 11-OH-THC is pharmacologically active as THC, and THC-COOH modulates THC’s effects [37]. THC interacts with various drug transporters and drug-metabolizing enzymes and may thereby alter the disposition of co-administered drugs, enhancing, in some cases, their adverse effects [37]. CBD, too, is metabolized by CYP450 enzymes to multiple metabolites [38]. Drug–drug interactions have been reported between CBD and other drugs. For example, the co-administration of CBD and the anti-epileptic drug clobazam leads to an increased metabolism of this drug, associated with an increase in adverse effects [38].

## 6. Concepts and Hallmarks of Cancer Stem Cells

A subpopulation of cancer stem cells (CSCs) can be identified in many malignant solid tumors. CSCs have been implicated in tumor initiation, progression, infiltration, and invasion, as well as tumor recurrence and metastasis, tumor angiogenesis, and chemotherapy resistance [39,40]; CSCs show resistance to radiotherapy and various types of conventional chemotherapy [40].

CSCs have relatively high clonogenic and tumorigenic potential and possess, similar to stem cells, a capacity for self-renewal through the self-generation of more stem cells. Importantly, CSCs are not discrete entities. Instead, a range of attributes in the CSC state is identified as a result of some CSC plasticity. In various cancer types, a “bidirectional interconversion” mode, i.e., the transition in daughter cells from stem to non-stem and non-stem to stem is constantly taking place [41]. Transition includes the de-differentiation of cancer cells towards stem-like phenotypes, ensuring the progression of cancer, and the multilineage differentiation of CSCs to increase genetic heterogeneity within the tumor mass [39,40,41,42]. Multilineage differentiation drives tumor growth and heterogeneity, and the heterogeneous, differentiated cancer cells form the bulk of the tumor [39,40,41,42]. Several different cell surface markers, including clusters of differentiation (CD) markers (e.g., CD24, CD133, and CD44), were shown in various cancers to be associated with CSC state and with aspect(s) of stem cell-like behavior [40,41,42,43,44]. 

Recent studies suggest that CSC plasticity allows them to differentiate into multiple phenotypic lineages and acquire alternate functions [45]. The tumor microenvironment (TME), including hypoxia-induced physical pressures, neighboring cell populations, exosomes, low pH, nutritional deficiencies, chemical signals, or inflammatory environments, was important in increasing CSC and neighboring cell plasticity. An example is vasculogenic mimicry (VM), a hallmark process by which cells in the vicinity of CSCs transdifferentiate to acquire endothelial cell-like properties [45]. TME induces non-CSCs to obtain CSC properties, altering the trajectory of the non-stem subpopulation on malignant tumor growth [41]. TME also increases immune escape by inducing immune or stromal cells to secrete cytokines and exosomes and to activate stemness pathways in CSCs [45]. In the case of triple-negative breast cancer, stemness pathways include Notch, JAK-STAT, Wnt/β-catenin, and Hedgehog, all of which are involved in CSC proliferation and differentiation [40,46]. These stemness-related signaling pathways are also involved with chemo-resistance processes in CSCs [40,47]. For example, increased expression of Notch3, a transmembrane receptor, is important for CSC platinum resistance, whereas the γ-secretase inhibitor (GSI) that inhibits Notch activity increases CSC platinum sensitivity [48]. In addition, activity on the Wnt pathway is correlated to cisplatin resistance in tumorigenic liver progenitor cells [49]. 

The Wnt pathway is inhibited by hypoxia-induced endoplasmic reticulum (ER) stress in human colorectal tumor cells [50]. ER stress is a series of cellular responses that lead to the disruption of ER homeostasis. In many cases, ER stress is induced by cellular Ca^2+^ overload and reactive oxygen species (ROS) accumulation and involves the accumulation of unfolded/misfolded proteins [51]. Various drug treatments induce ER stress in solid tumors [51]. ER stress reduces the population and invasion of stem cell-like cancer cells. This was demonstrated on a CD44+/CD24− subpopulation of breast cancer cells following tunicamycin treatment [52]. However, in another case, it was suggested that intercellular signaling leading to transmissible ER stress (TERS) induces Wnt signaling in recipient human prostate cancer cells [53].

Some CSC populations are also characterized by epithelial–mesenchymal transition (EMT). EMT is the change from epithelial to mesenchymal cellular phenotypes. These phenotypic changes are associated with the high expression of vimentin and N-cadherin. EMT plays a role in cell plasticity, intra-tumor heterogeneity, and cell migration, among other properties [54]. 

Several aspects link the EMT phenotype and CSC state. One aspect relates to the genetic pathways altered in both the CSC and EMT, including the MAPK/ERK, JAK/STAT, TGFβ-SMAD, Wnt/β-catenin, and PI3K-AKT-NFκB pathways [54,55]. The EMT is also linked to CSCs in immune modulation, including the resistance to cytotoxic T lymphocytes and the presence of tumor-associated macrophages in both the EMT phenotype and CSC states [54,56]. Acquiring EMT phenotypes in CSCs probably promotes the metastatic proliferation of these cells [54]. The EMT can induce cancer cells that are non-tumorigenic into a CSC-like state, whereas CSCs can modulate their niche for maintaining EMT homeostasis [54]. 

## 7. Ovarian Cancer Stem Cells and Drug Resistance Mechanisms

Spheroids are often found within the peritoneal ascites associated with OC tumors. These spheres survive and proliferate in a substrate non-adherent status [47]. Cells sorted for positive CSC markers such as CD44+/CD117+ and CD133+ were designated as stem-like cells of ovarian cancer (ovarian cancer stem cells; OCSC) and were found to have a higher ability to form spheres and higher tumorigenesis compared to cells without CSC markers [47,57]. 

CD44 is a cell surface receptor, an integral membrane glycoprotein that binds several extracellular matrix (ECM) components, including hyaluronan [58], and which plays a diverse role in ovarian cancer progression, cell proliferation, migration, invasion, and metastasis [44]. The chemotherapy resistance displayed by CD44-positive OCSCs is suggested to be due to the expression of myeloid differentiation factor 88 (MyD88), which leads to the activation of the nuclear factor kappa B (NF-κB) signaling pathway and the production of various cytokines (Table 2; [59]). NF-κB signaling is suggested to promote intratumoral heterogeneity and, in OCSCs, to contribute to chemotherapy drug resistance [44]. 

CD117 (c-Kit), involved in cellular survival and cancer cell differentiation, is a type III tyrosine kinase receptor, a member of the platelet-derived growth factor receptor subfamily [44]. CD117, via activation of the PI3K/AKT and Wnt/β-catenin signaling pathways, is associated with increased expression of ATP-binding cassette subfamily G member 2 (ABCG2), an ABC transporter [60]. The Wnt/β-catenin pathway also induces aldehyde dehydrogenase (ALDH) 1A1 activity in platinum-resistant OC cells [44,61]. High ALDH activity is associated with OCSC marker expression, self-renewal, colony and tumor formation, and EMT processes [47]. ALDH likely confers drug resistance by enhancing drug metabolism (Table 2). For example, ALDH metabolizes cyclophosphamide, an alkylating agent used against leukemic stem cells, to the inactive excretory product 4-hydroperoxycyclophosphamide, conferring specific drug resistance to these cells [62]. Similarly, CD117 has been demonstrated to mediate OCSC chemotherapy resistance to paclitaxel and cisplatin [60] and platinum resistance in OC patient-derived xenograft cells by exhibiting a marked increase in the expression of certain Wnt/β-catenin target genes [61]. The expression of several cancer stem cell markers, including ALDH1A1, is also enhanced in these OC patient-derived platinum-resistant xenograft cells [61]. 

Another drug-resistance mechanism in OCSCs is associated with the increased expression of ATP-binding cassette (ABC) transporters. In some chemotherapy-resistant cancer cells, ABC transporters pump various chemotherapies out of the cell, such as doxorubicin and paclitaxel (Table 2). In particular, ABC subfamily A member 1 (ABCA1), ABC subfamily B member 1 (ABCB1/MDR1/P-GP), and ABC subfamily G member 2/breast cancer resistance protein (ABCG2/BCRP) are highly expressed in OCSCs [47]. Hedgehog-GLI (HH-GLI) is involved in the upregulation of ABCB1 and ABCG2 gene expression via the Gli1-BMI-1 signaling pathway. The activity of HH-GLI is closely associated with cisplatin resistance in OC cells [44]. 

As described above, the marker CD133, a member of the pentaspan transmembrane protein family, is positive in OCSCs [44,57]. The expression and display of CD133 in colorectal cancer cells is associated with the upregulation of an inhibitor of DNA binding (ID)1 proteins expression (Table 2; [63]). ID proteins are highly conserved transcriptional regulators. They are essential components of oncogenic pathways and maintain self-renewal and multipotency in stem cells while inhibiting their differentiation [64]. It was shown that knockdown of ID1 impairs cell proliferation and sphere formation capacity and reverses EMT-associated traits. It was also suggested that ID1 maintains colorectal cancer stemness partially via the Wnt/β-catenin signaling pathway [63].

Activity of the B-cell lymphoma-2 (BCL-2) protein family, potential oncogenes, is another mechanism that may lead to chemo-resistance in OCSCs (Table 2; [47]). Overexpression of Bcl-xL, a member of the BCL-2 protein family, has been observed in most recurrent chemo-resistant ovarian cancers, and inhibition of Bcl-xL in preclinical studies increased the chemo-sensitivity of OC cells [47]. These proteins inhibit the activation of the BAX and BAK pro-apoptotic proteins [47]. BAX and BAK, upon activation, are converted from inert monomers into oligomers that lead to membrane permeability and the release of cytochrome c (cyt c) from mitochondria. The release of cyt c induces caspase activity and ensures apoptosis [65].

## 8. Studies That Have Examined the Effectivity of Cannabis Compounds against OC

### 8.1. Preclinical

Only a few studies have examined the effectivity of phytocannabinoids against OC. In an OC cell line and in a chick embryo model (i.e., in ovo), CBD was shown to have anti-proliferative activity [66]. The administration of CBD carried by nanoparticles or in solution also increased paclitaxel treatment effectivity in vitro and in ovo [66,67]. However, there is no peer-reviewed information on the effect of cannabis and cannabis compounds on OCSC development or properties.

### 8.2. A Single Patient Case Study and Epidemiological Overview

Only a single patient case study has been published in the scientific literature. This study demonstrated that treatment of a low-grade serous ovarian cancer patient with “CBD oil” improved the expression of markers associated with the disease [68]. Nevertheless, multiple compounds might be present in the “CBD oil” since, in many cases, “CBD oils” are full extracts of high CBD cannabis inflorescence (notably, this information is not provided in [68]). As a result, the actual cannabis molecules and their combination(s) that might be active against OC markers have not been identified. In contrast, an epidemiological overview and survey of cannabis and phytocannabinoid users in the USA between 2003 and 2017 on the occurrence of prostate and ovarian cancers suggested that CBD might be considered a community carcinogen, additive to the effects of tobacco [69]. 

## 9. Preclinical Evidence on the Cannabis Mode of Action on Genetic Pathways Related to OCSC

Since there is no peer-reviewed information on the effect of cannabis and cannabis compounds on OCSC development and properties, we sought to summarize the known effects of phytocannabinoids on genetic pathways and biological processes related to CSCs and OCSCs. Indeed, several of the major phytocannabinoids have been shown to affect signaling pathways that are mainly involved with OC stemness. 

**Figure 1:**
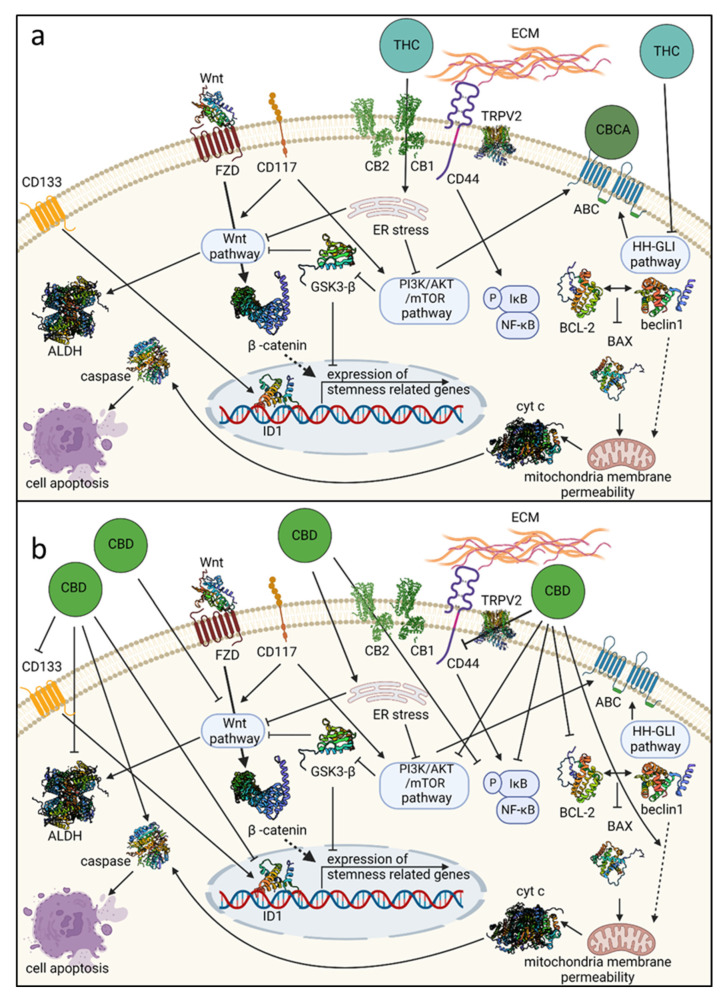
Some of the genetic pathways and mechanisms affected by phytocannabinoids: THC and CBCA (**a**) and CBD (**b**) that are associated with ovarian cancer stem cell state. Receptors are illustrated in cases where receptor involvement in activity was suggested. ABC, ATP-binding cassette transporter; ALDH, aldehyde dehydrogenase; BCL-2, the activity of B-cell lymphoma-2; CB1, cannabinoid receptor type 1; CB2, cannabinoid receptor type 2; CBCA, cannabichromenic acid; CBD, cannabidiol; CD, clusters of differentiation; cyt c, cytochrome c; ECM, extracellular matrix; ER stress, endoplasmic reticulum stress; FZD, Wnt frizzled receptor; HH-GLI, Hedgehog-GLI; ID1, an inhibitor of DNA binding; THC, Δ^9^-trans-tetrahydrocannabinol; TRPV2, transient receptor potential cation channel subfamily V member 2. Created with BioRender.com (accessed on 29 August 2022).

CBD, cannabidivarin (CBDV; Table 1), and the 2,3-epoxy derivative of CBD exhibited, in a dose-dependent manner, considerable inhibitory activity against the Wnt/β-catenin pathway [70]. In general, enhanced Wnt/β-catenin pathway activity is a hallmark of the stemness and drug resistance of OCSC and other cancers [46,47,49]. In addition, in vivo intraperitoneal administration of “cannabis smoke” (in the form of condensate cannabis resin extract collected in a smoking machine and dissolved in olive oil) inhibited ALDH activity in a rat model [71]. ALDH activity, induced by the Wnt/β-catenin pathway, is another marker closely associated with the OCSC state [44,47,61]. In this case, it might be that inhibition of the Wnt/β-catenin signaling pathway promoted the inhibition of ALDH activity (Figure 1). 

THC (via CB1 activation) and CBD were shown to inhibit Akt phosphorylation in colorectal cancer cells and to induce apoptosis in these cells via inhibition of the PI3K-Akt survival signaling cascade (Figure 1; [72,73]). In contrast, CBD and THC were suggested to promote PI3K/Akt signaling and thereby downregulate the expression of GSK3-β, a serine/threonine kinase and an inhibitor of the Wnt/β-catenin pathway in various glaucoma-related models [74]. However, these trends of upregulation of PI3K/Akt signaling by CBD or THC were primarily shown in non-cancerous cells (e.g., mesenchymal stem cells derived from gingiva; non-cancerous mice brain [74]) and are opposite to what was reported for cancer cells [72,73]. Perhaps the activity of CBD or THC on malignant and non-malignant cells differs with their effect on the PI3K/Akt and related pathways.

As detailed above, the HH-GLI signaling pathway is involved with OC and OCSC drug resistance and upregulation of the ABC transporters’, ABCB1 and ABCG2, gene expression [44]. THC is a direct inhibitor of the HH-GLI signaling pathway in vitro, and in mice that bear a subthreshold defect in HH signaling. The inhibitory activity of the HH-GLI pathway by THC was not mediated via the CB1 receptor (Figure 1; [75]). HH-GLI pathway inhibition by phytocannabinoids may suggest the alteration of ABC transporter expression and activity by these compounds. Increased sensitivity to THC-induced hypothermia was evident in ABC transporter knockout mice in comparison to wild type mice [76], further solidifying a connection between the THC and ABC transporter expression or activity. In contrast, using the in vitro bidirectional transport assay, it was shown that neither CBCA nor CBC inhibited the ABC transporter activity. CBCA only served as a substrate for the ABCB1 transporter (Figure 1; [77]). CBD is also not a substrate of the ABC transporters ABCB1 or ABCG2 [78].

CBD treatment downregulated the expression of CD44 in human gingival mesenchymal stem cells in vitro (Figure 1; [79]). A reduction in the number of CD44+ and CD133+ cells was also obtained with CBD treatment of cisplatin-resistant non-small cell lung cancer compared to controls. This treatment suppressed additional CSC-associated properties, including sphere formation and protein expression of Snail, Nanog, and Vimentin in cell lines [80]. The apoptotic activity of CBD on the cell lines was mediated via the TRPV2 receptor. In addition, in a mouse xenograft model of these drug-resistant non-small cell lung cancer cells, CBD treatment reduced tumor progression and metastasis [80]. 

In lipopolysaccharide (LPS)-stimulated microglia cells, CBD inhibited NADPH oxidase-mediated ROS production and NF-κB-dependent signaling events (Figure 1; [81]). This activity was only slightly reduced by blocking the CB2 receptor and was mainly receptor-independent [81]. This inhibitory activity of CBD on NF-κB-dependent signaling events might accord with its inhibitory effect on CD44+ expression or display, as the NF-κB pathway is activated in CD44+ OCSCs [44,59]. 

CBD and other phytocannabinoids induce endoplasmic reticulum stress [31,32,82] and, subsequently, in MDA-MB231 breast cancer cells, inhibit AKT and mTOR signaling (Figure 1; [82]). In these cells, CBD inhibited the association between BCL-2 and beclin1 and induced Bax elevation (Figure 1; [82]). It also activated caspase-8 and led to the cleavage of beclin-1 [82]. This cleavage product translocases to mitochondria and enhances the release of cyt c to the cytosol [82]. In support, in cisplatin-resistant non-small cell lung cancer cells, an increase in cleaved caspase-3 and cyt c release was evident upon CBD treatments [80]. As indicated above, the release of cyt c to the cytoplasm activates caspase and ensures apoptosis (Figure 1; [65,82]). 

CBD was demonstrated to inhibit the expression of ID1 in breast cancer cells, which maintains cancer stemness [64], and, as a direct result, leads to anti-metastatic activity [83]. CBD also inhibited the expression of ID1 in head and neck, prostate, and salivary gland cancers [84]. In vivo, mice with advanced metastatic progression, once treated with the CBD analog O-1663, had a high survival rate, even beyond that of the group treated with CBD [13,83]. 

Lastly, CBD was shown to inhibit EMT and induce reversion to a non-invasive phenotype in breast cancer cells [33]. CBD treatment blocked cell migration and inhibited the progression of the IL-1β/IL-1RI/β-catenin signaling pathway [33]. The treatment re-localized β-catenin and E-cadherin to the adherent junctions and re-established the epithelial organization lost by the IL-1β-induced dispersion of the cells. It also prevented the nuclear translocation of β-catenin and decreased the overexpression of several genes, ID1 proteins included [33]. Here too, CBD treatment inhibited AKT activation (Figure 1; [33]). 

## 10. Conclusions

It is essential to find new means to fight OC, as it is the second most common—and the most lethal—gynecologic malignancy in the western world. OCSCs are implicated in tumor recurrence, metastasis, and drug resistance, and it is clear that this subpopulation of cells should be targeted specifically. To date, many commonly used chemotherapies or monotherapies stimulate, rather than reduce, this cell subpopulation. For example, PARP inhibitors (PARPi) are monotherapy agents approved by the FDA for the treatment of recurrent OC in patients with or without a BRCA mutation. Unfortunately, PARPi treatment induced the enrichment of CD117+ and CD133+ OCSCs in vitro and in vivo, regardless of BRCA mutation status [85]. OCSCs activate embryonic repair mechanisms, which increase DNA repair efficiency in these malignant cells [85]. As a result, it was suggested that PARPi treatment could fail to significantly affect OCSC populations and might lead instead to difficulties in reducing recurrent OC [85]. Similarly, CD44+ OCSCs survive carboplatin treatment via the activation of NF-κB and PI3K/AKT signaling pathways [86]. It was suggested that a rational approach to prevent platinum-resistant relapse is by perturbing CSC homeostasis, e.g., by blocking PI3K/AKT signaling [86]. From these few examples, it is clear that the unique state of OCSC needs to be examined, and approaches to target OCSC specifically might be promising. 

Although very little is known on the activity of phytocannabinoids against OC in general and OCSCs in particular, phytocannabinoids affect hallmark signaling pathways and functional markers that are intimately associated with OCSC, such as the Wnt/β-catenin pathway, ALDH activity, PI3K/Akt, HH-GLI- and NF-κB-dependent signaling pathways, and BCL2- and ID1-related processes. It is reasonable to suggest that chemotherapy or monotherapy treatments could be combined with phytocannabinoid(s) to reduce recurring OC and resistance relapse. 

As detailed above, cannabis produces more than 150 phytocannabinoids, including the abundant CBD and THC with well-known anti-cancer activities. However, other phytocannabinoids might possess anti-cancer activity, and it has been shown in other studies that combination(s) of multiple compounds can improve the beneficial effects [87]. THC binds both to CB1 and CB2 receptors as an agonist, while CBD binds CB1 as an antagonist, but there are no biochemical indications of the physical association between other phytocannabinoids and receptors. On the other hand, most THC and CBD activity on the CSC-related pathways is not clearly or fully mediated via known CB1 or CB2 (or other) receptors, as detailed above. This leaves the possibility that other phytocannabinoids might be related to anti-CSC activity, via various other cell membrane receptors or by intracellular activities. 

The activity of several molecules from cannabis has been suggested to be superior vs. that of a single molecule. This phenomenon was named the “entourage effect” [87,88,89] and we recognize today that at least part of the entourage effect is a result of synergy between cannabis molecules [87]. This synergy might be a result of the co-activation of several receptors by the various molecules, but could also be the result of co-affecting several signaling pathways, leading to an increased response [87,90]. 

For example, it was shown in OC cells (detailed above) that THC affects the HH-GLI pathway, which may reduce the expression/activity of ABC transporters (Figure 1), and thereby (potentially) reduce resistance to chemotherapy such as cisplatin. Likewise, CBD represses PI3K/AKT signaling (Figure 1), which raises cellular sensitivity to cisplatin. In this example, it might be beneficial to co-treat with THC and/or CBD and cisplatin for increased sensitivity to the chemotherapy agent. 

The complementary activity of chemotherapy agents and phytomolecules should be further explored in preclinical and clinical trials. Optimizing phytocannabinoid-based therapies necessitates an increased understanding of the molecular mechanisms and the receptors involved in phytocannabinoid anti-tumor activity, as well as designing and testing in preclinical models the most effective phytocannabinoid combinations, with or without chemotherapy or monotherapy. Finally, conducting controlled studies on cancer patients is essential and so far unrealized [13,87].

A note should be given to the publication [69] that suggested, based on an epidemiological overview and survey of USA cannabis and cannabinoid users, that CBD might be considered a community carcinogen, additive to the effects of tobacco. Due to the considerable effect of CBD on cancer-associated genetic pathways, adverse effects from activating these pathways are possible. To avoid adverse effects and to better control cannabis medical use, cannabis and CBD-based products should be administered with known and controlled compositions and dosage [87].

Yet, THC, CBD, and cannabis are used widely for medical purposes today. Cannabis and cannabis compounds are common palliative treatments for cancer patients, including anti-nausea and vomiting treatment associated with chemotherapy, appetite stimulation, and cancer-related pain relief [87]. Rationally, since these compounds are already widely medically used, they could be combined with chemotherapy or monotherapy relatively easily (e.g., regulatory-wise). The potential benefit of these combined treatments should be examined.

## Figures and Tables

**Table 1:** Representative structures of the relevant phytocannabinoids.

Phytocannabinoid	Chemical Structure
THCA/THC	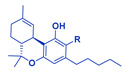
CBDA/CBD	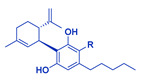
CBGA/CBG	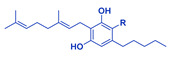
CBDVA/CBDV	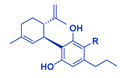
CBCA/CBC	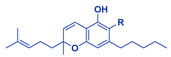

Abbreviations: CBDA, cannabidiolic acid; CBD, cannabidiol; CBGA, cannabigerolic acid; CBG, cannabigerol; THCA, Δ^9^-tetrahydrocannabinolic acid; THC, Δ^9^-tetrahydrocannabinol; CBDVA, cannabidivarinic acid; CBDV, cannabidivarin; CBCA, cannabichromenic acid; CBC, cannabichromene. R=COOH in the case of acids. R=H following decarboxylation. Chemical structures were drawn by Dr. Seegehalli M Anil.

**Table 2:** Ovarian cancer stem cells and drug resistance mechanisms.

Marker/Protein	Suggested Drug Resistance Mechanism(s)	**References**
CD44, a cell surface receptor, an integral membrane glycoprotein that binds several ECM components, including hyaluronan	Activation of NF-κB signaling pathway and the production of various cytokines	[44,58,59]
CD117 (c-Kit), a type III tyrosine kinase receptor	Activation of PI3K/AKT and Wnt/β-catenin signaling pathway and increased expression of ABC transporters.	[44,47,60,61]
ABC transporters	ABC transporters pump out of the cell various chemotherapies.	[44,47,60]
ALDH, aldehyde dehydrogenase	Enhanced drug metabolism.	[44,47,61,62]
CD133, a member of the pentaspan transmembrane protein family	Expression of ID1 proteins transcriptional regulators.	[44,57,63,64]
Bcl-xL, BCL-2 protein family	Inhibition of the activation of the BAX and BAK pro-apoptotic proteins.	[47,65]

## Appendix A

Methodology: To summarize and discuss the hallmarks of CSC and OCSC, and the effect of cannabis and phytocannabinoids on OC, CSC, OCSC, and biological pathways associated with stemness, we conducted a literature review using the following terms: “cancer“, “ovarian cancer”, “stem cells”, “ovarian cancer stem cells”, “medical use of cannabis”, “therapy” “*Cannabis sativa*”, “*C. sativa*”, “cannabis”, “cannabinoids”, “phytocannabinoids”, “cannabis oil”, “endocannabinoid”, “entourage effect”, “stemness”, “epithelial-mesenchymal transition”, “ER stress”, “MAPK/ERK”, “JAK/STAT”, “Wnt/β-catenin”, and “PI3K-AKT-NFκB”. The search was conducted on general and multidisciplinary research databases for peer-reviewed scientific manuscripts, including PubMed, Google Scholar, Scopus, and Web of Science.

## References

[1] Cortez A.J., Tudrej P., Kujawa K.A., Lisowska K.M. (2018). Advances in ovarian cancer therapy. Cancer Chemother. Pharmacol..

[2] Ottevanger P.B. (2017). Ovarian Cancer Stem Cells More Questions than Answers. Seminars in Cancer Biology.

[3] Menon U., Gentry-Maharaj A., Burnell M., Singh N., Ryan A., Karpinskyj C., Carlino G., Taylor J., Massingham S.K., Raikou M. (2021). Ovarian cancer population screening and mortality after long-term follow-up in the UK Collaborative Trial of Ovarian Cancer Screening (UKCTOCS): A randomised controlled trial. Lancet.

[4] Barnett R. (2016). Ovarian cancer. Lancet.

[5] Radu M.R., Prădatu A., Duică F., Micu R., Creţoiu S.M., Suciu N., Creţoiu D., Varlas V.N., Rădoi V.E. (2021). Ovarian cancer: Biomarkers and targeted therapy. Biomedicines.

[6] Li H., Liu Y., Wang Y., Zhao X., Qi X. (2021). Hormone therapy for ovarian cancer: Emphasis on mechanisms and applications. Oncol. Rep..

[7] Corroon J., Sexton M., Bradley R. (2019). Indications and administration practices amongst medical cannabis healthcare providers: A cross-sectional survey. BMC Fam. Pract..

[8] Hanuš L.O., Meyer S.M., Muñoz E., Taglialatela-Scafati O., Appendino G. (2016). Phytocannabinoids: A unified critical inventory. Nat. Prod. Rep..

[9] Gülck T., Møller B.L. (2020). Phytocannabinoids: Origins and biosynthesis. Trends Plant Sci..

[10] Aizpurua-Olaizola O., Soydaner U., Öztürk E., Schibano D., Simsir Y., Navarro P., Etxebarria N., Usobiaga A. (2016). Evolution of the cannabinoid and terpene content during the growth of *Cannabis sativa* plants from different chemotypes. J. Nat. Prod..

[11] Ramer R., Hinz B. (2017). Cannabinoids as anticancer drugs. Adv. Pharmacol..

[12] Velasco G., Sánchez C., Guzmán M. (2012). Towards the use of cannabinoids as antitumour agents. Nat. Rev. Cancer.

[13] McAllister S.D., Abood M.E., Califano J., Guzmán M. (2021). Cannabinoid cancer biology and prevention. J. Natl. Cancer Inst..

[14] Bautista J.L., Yu S., Tian L. (2021). Flavonoids in *Cannabis sativa*: Biosynthesis, bioactivities, and biotechnology. ACS Omega.

[15] Tahir M.N., Shahbazi F., Rondeau-Gagné S., Trant J.F. (2021). The biosynthesis of the cannabinoids. J. Cannabis Res..

[16] Di Marzo V., Piscitelli F. (2015). The endocannabinoid system and its modulation by phytocannabinoids. Neurotherapeutics.

[17] Hillard C.J. (2018). Circulating endocannabinoids: From whence do they come and where are they going?. Neuropsychopharmacology.

[18] Laezza C., Pagano C., Navarra G., Pastorino O., Proto M.C., Fiore D., Piscopo C., Gazzerro P., Bifulco M. (2020). The endocannabinoid system: A target for cancer treatment. Int. J. Mol. Sci..

[19] Hinz B., Ramer R. (2019). Anti-tumour actions of cannabinoids. Br. J. Pharmacol..

[20] Kovalchuk O., Kovalchuk I. (2020). Cannabinoids as anticancer therapeutic agents. Cell Cycle.

[21] Taylor A.H., Tortolani D., Ayakannu T., Konje J.C., Maccarrone M. (2020). (Endo) cannabinoids and gynaecological cancers. Cancers.

[22] Fraguas-Sánchez A.I., Martín-Sabroso C., Torres-Suárez A.I. (2018). Insights into the effects of the endocannabinoid system in cancer: A review. Br. J. Pharmacol..

[23] Abood M., Alexander S.P., Barth F., Bonner T.I., Bradshaw H., Cabral G., Casellas P., Cravatt B.F., Devane W.A., Di Marzo V. (2019). Cannabinoid receptors (version 2019.4) in the IUPHAR/BPS Guide to Pharmacology Database. IUPHAR/BPS Guide Pharmacol. CITE.

[24] Maccarrone M. (2020). Phytocannabinoids and endocannabinoids: Different in nature. Rend. Lincei. Sci. Fis. Nat..

[25] Biringer R.G. (2021). Endocannabinoid signaling pathways: Beyond CB1R and CB2R. J. Cell Commun. Signal.

[26] Duggan P.J. (2021). The Chemistry of cannabis and cannabinoids. Aust. J. Chem..

[27] Tomko A.M., Whynot E.G., Ellis L.D., Dupré D.J. (2020). Anti-cancer potential of cannabinoids, terpenes, and flavonoids present in cannabis. Cancers.

[28] Velasco G., Hernández-Tiedra S., Dávila D., Lorente M. (2016). The use of cannabinoids as anticancer agents. Prog. Neuropsychopharmacol. Biol. Psychiatry.

[29] Blázquez C., Salazar M., Carracedo A., Lorente M., Egia A., González-Feria L., Haro A., Velasco G., Guzmán M. (2008). Cannabinoids inhibit glioma cell invasion by down-regulating matrix metalloproteinase-2 expression. Cancer Res..

[30] Carracedo A., Lorente M., Egia A., Blázquez C., García S., Giroux V., Malicet C., Villuendas R., Gironella M., González-Feria L. (2006). The stress-regulated protein p8 mediates cannabinoid-induced apoptosis of tumor cells. Cancer Cell.

[31] Peeri H., Shalev N., Vinayaka A.C., Nizar R., Kazimirsky G., Namdar D., Anil S.M., Belausov E., Brodie C., Koltai H. (2021). Specific Compositions of *Cannabis sativa* Compounds Have Cytotoxic Activity and Inhibit Motility and Colony Formation of Human Glioblastoma Cells In Vitro. Cancers.

[32] Peeri H., Koltai H. (2022). Cannabis biomolecule effects on cancer cells and cancer stem cells: Cytotoxic, anti-proliferative, and anti-migratory activities. Biomolecules.

[33] García-Morales L., Castillo A.M., Tapia Ramírez J., Zamudio-Meza H., Domínguez-Robles M.d.C., Meza I. (2020). CBD reverts the mesenchymal invasive phenotype of breast cancer cells induced by the inflammatory cytokine IL-1β. Int. J. Mol. Sci..

[34] Jacobsson S.O., Rongård E., Stridh M., Tiger G., Fowler C.J. (2000). Serum-dependent effects of tamoxifen and cannabinoids upon C6 glioma cell viability. Biochem. Pharmacol..

[35] Ligresti A., Moriello A.S., Starowicz K., Matias I., Pisanti S., De Petrocellis L., Laezza C., Portella G., Bifulco M., Di Marzo V. (2006). Antitumor activity of plant cannabinoids with emphasis on the effect of cannabidiol on human breast carcinoma. J. Pharmacol. Exp. Ther..

[36] Anis O., Vinayaka A.C., Shalev N., Namdar D., Nadarajan S., Anil S.M., Cohen O., Belausov E., Ramon J., Mayzlish Gati E. (2021). Cannabis-derived compounds cannabichromene and Δ9-tetrahydrocannabinol interact and exhibit cytotoxic activity against urothelial cell carcinoma correlated with inhibition of cell migration and cytoskeleton organization. Molecules.

[37] Brown J.D. (2020). Potential adverse drug events with tetrahydrocannabinol (THC) due to drug–drug interactions. J. Clin. Med..

[38] Alves P., Amaral C., Teixeira N., Correia-da-Silva G. (2020). *Cannabis sativa*: Much more beyond Δ9-tetrahydrocannabinol. Pharmacol. Res..

[39] Jordan C.T., Guzman M.L., Noble M. (2006). Cancer stem cells. N. Engl. J. Med..

[40] Zhou H.-M., Zhang J.-G., Zhang X., Li Q. (2021). Targeting cancer stem cells for reversing therapy resistance: Mechanism, signaling, and prospective agents. Signal Transduct. Target Ther..

[41] Heft Neal M.E., Brenner J.C., Prince M.E.P., Chinn S.B. (2022). Advancement in cancer stem cell biology and precision medicine-review article, head and neck cancer stem cell plasticity and the tumor microenvironment. Front. Cell Dev. Biol..

[42] Ayob A.Z., Ramasamy T.S. (2018). Cancer stem cells as key drivers of tumour progression. J. Biomed. Sci..

[43] He L., Wick N., Germans S.K., Peng Y. (2021). The role of breast cancer stem cells in chemoresistance and metastasis in triple-negative breast cancer. Cancers.

[44] Motohara T., Yoshida G.J., Katabuchi H. (2021). The hallmarks of ovarian cancer stem cells and niches: Exploring their harmonious interplay in therapy resistance. Semin. Cancer Biol..

[45] Zheng X., Yu C., Xu M. (2021). Linking tumor microenvironment to plasticity of cancer stem cells: Mechanisms and application in cancer therapy. Front. Oncol..

[46] Qayoom H., Wani N.A., Alshehri B., Mir M.A. (2021). An insight into the cancer stem cell survival pathways involved in chemoresistance in triple-negative breast cancer. Future Oncol..

[47] Keyvani V., Farshchian M., Esmaeili S.-A., Yari H., Moghbeli M., Nezhad S.-R.K., Abbaszadegan M.R. (2019). Ovarian cancer stem cells and targeted therapy. J. Ovarian Res..

[48] McAuliffe S.M., Morgan S.L., Wyant G.A., Tran L.T., Muto K.W., Chen Y.S., Chin K.T., Partridge J.C., Poole B.B., Cheng K.-H. (2012). Targeting Notch, a key pathway for ovarian cancer stem cells, sensitizes tumors to platinum therapy. Proc. Natl. Acad. Sci. USA.

[49] Yang W., Yan H.-X., Chen L., Liu Q., He Y.-Q., Yu L.-X., Zhang S.-H., Huang D.-D., Tang L., Kong X.-N. (2008). Wnt/β-catenin signaling contributes to activation of normal and tumorigenic liver progenitor cells. Cancer Res..

[50] Verras M., Papandreou I., Lim A.L., Denko N.C. (2008). Tumor hypoxia blocks Wnt processing and secretion through the induction of endoplasmic reticulum stress. Mol. Cell. Biol..

[51] Cao S., Tang J., Huang Y., Li G., Li Z., Cai W., Yuan Y., Liu J., Huang X., Zhang H. (2021). The road of solid tumor survival: From drug-induced endoplasmic reticulum stress to drug resistance. Front. Mol. Biosci..

[52] Nami B., Donmez H., Kocak N. (2016). Tunicamycin-induced endoplasmic reticulum stress reduces in vitro subpopulation and invasion of CD44+/CD24-phenotype breast cancer stem cells. Exp. Toxicol. Pathol..

[53] Rodvold J.J., Chiu K.T., Hiramatsu N., Nussbacher J.K., Galimberti V., Mahadevan N.R., Willert K., Lin J.H., Zanetti M. (2017). Intercellular transmission of the unfolded protein response promotes survival and drug resistance in cancer cells. Sci. Signal..

[54] Tanabe S., Quader S., Cabral H., Ono R. (2020). Interplay of EMT and CSC in cancer and the potential therapeutic strategies. Front. Pharmacol..

[55] Loret N., Denys H., Tummers P., Berx G. (2019). The role of epithelial-to-mesenchymal plasticity in ovarian cancer progression and therapy resistance. Cancers.

[56] Terry S., Chouaib S. (2015). EMT in immuno-resistance. Oncoscience.

[57] Klemba A., Purzycka-Olewiecka J.K., Wcisło G., Czarnecka A.M., Lewicki S., Lesyng B., Szczylik C., Kieda C. (2018). Surface markers of cancer stem-like cells of ovarian cancer and their clinical relevance. Contemp. Oncol./Współczesna Onkol..

[58] Bourguignon L.Y. (2019). Matrix hyaluronan-CD44 interaction activates MicroRNA and LncRNA signaling associated with chemoresistance, invasion, and tumor progression. Front. Oncol..

[59] Alvero A.B., Chen R., Fu H.-H., Montagna M., Schwartz P.E., Rutherford T., Silasi D.-A., Steffensen K.D., Waldstrom M., Visintin I. (2009). Molecular phenotyping of human ovarian cancer stem cells unravels the mechanisms for repair and chemoresistance. Cell Cycle.

[60] Chau W., Ip C., Mak A., Lai H., Wong A. (2013). c-Kit mediates chemoresistance and tumor-initiating capacity of ovarian cancer cells through activation of Wnt/β-catenin–ATP-binding cassette G2 signaling. Oncogene.

[61] Nagaraj A.B., Joseph P., Kovalenko O., Singh S., Armstrong A., Redline R., Resnick K., Zanotti K., Waggoner S., DiFeo A. (2015). Critical role of Wnt/β-catenin signaling in driving epithelial ovarian cancer platinum resistance. Oncotarget.

[62] Hilton J. (1984). Role of aldehyde dehydrogenase in cyclophosphamide-resistant L1210 leukemia. Cancer Res..

[63] Sun Y., Lai X., Yu Y., Li J., Cao L., Lin W., Huang C., Liao J., Chen W., Li C. (2019). Inhibitor of DNA binding 1 (Id1) mediates stemness of colorectal cancer cells through the Id1-c-Myc-PLAC8 axis via the Wnt/β-catenin and Shh signaling pathways. Cancer Manag. Res..

[64] Lasorella A., Benezra R., Iavarone A. (2014). The ID proteins: Master regulators of cancer stem cells and tumour aggressiveness. Nat. Rev. Cancer.

[65] Moldoveanu T., Czabotar P.E. (2020). BAX, BAK, and BOK: A coming of age for the BCL-2 family effector proteins. Cold Spring Harb. Perspect. Biol..

[66] Fraguas-Sánchez A.I., Torres-Suárez A.I., Cohen M., Delie F., Bastida-Ruiz D., Yart L., Martin-Sabroso C., Fernández-Carballido A. (2020). PLGA nanoparticles for the intraperitoneal administration of CBD in the treatment of ovarian cancer: In Vitro and In Ovo assessment. Pharmaceutics.

[67] Fraguas-Sánchez A., Fernández-Carballido A., Delie F., Cohen M., Martin-Sabroso C., Mezzanzanica D., Figini M., Satta A., Torres-Suárez A. (2020). Enhancing ovarian cancer conventional chemotherapy through the combination with cannabidiol loaded microparticles. Eur. J. Pharm. Biopharm..

[68] Barrie A.M., Gushue A.C., Eskander R.N. (2019). Dramatic response to Laetrile and cannabidiol (CBD) oil in a patient with metastatic low grade serous ovarian carcinoma. Gynecol. Oncol. Rep..

[69] Reece A.S., Hulse G.K. (2022). Geotemporospatial and causal inferential epidemiological overview and survey of USA cannabis, cannabidiol and cannabinoid genotoxicity expressed in cancer incidence 2003–2017: Part 3–spatiotemporal, multivariable and causal inferential pathfinding and exploratory analyses of prostate and ovarian cancers. Arch. Public Health.

[70] Nalli Y., Dar M.S., Bano N., Rasool J.U., Sarkar A.R., Banday J., Bhat A.Q., Rafia B., Vishwakarma R.A., Dar M.J. (2019). Analyzing the role of cannabinoids as modulators of Wnt/β-catenin signaling pathway for their use in the management of neuropathic pain. Bioorg. Med. Chem. Lett..

[71] Marselos M., Vasiliou V., Malamasi M., Alikaridis F., Kefalas T. (1991). Effects of cannabis and tobacco on the enzymes of alcohol metabolism in the rat. Rev. Environ. Health.

[72] Greenhough A., Patsos H.A., Williams A.C., Paraskeva C. (2007). The cannabinoid δ9-tetrahydrocannabinol inhibits RAS-MAPK and PI3K-AKT survival signalling and induces BAD-mediated apoptosis in colorectal cancer cells. Int. J. Cancer.

[73] Aviello G., Romano B., Borrelli F., Capasso R., Gallo L., Piscitelli F., Di Marzo V., Izzo A.A. (2012). Chemopreventive effect of the non-psychotropic phytocannabinoid cannabidiol on experimental colon cancer. J. Mol. Med..

[74] Vallée A., Lecarpentier Y., Vallée J.-N. (2021). Cannabidiol and the canonical WNT/β-catenin pathway in glaucoma. Int. J. Mol. Sci..

[75] Lo H.-F., Hong M., Szutorisz H., Hurd Y.L., Krauss R.S. (2021). Δ9-tetrahydrocannabinol inhibits Hedgehog-dependent patterning during development. Development.

[76] Spiro A.S., Wong A., Boucher A.A., Arnold J.C. (2012). Enhanced brain disposition and effects of Δ9-tetrahydrocannabinol in P-glycoprotein and breast cancer resistance protein knockout mice. PLoS ONE.

[77] Etchart M.G., Anderson L.L., Ametovski A., Jones P.M., George A.M., Banister S.D., Arnold J.C. (2022). In vitro evaluation of the interaction of the cannabis constituents cannabichromene and cannabichromenic acid with ABCG2 and ABCB1 transporters. Eur. J. Pharmacol..

[78] Brzozowska N., Li K.M., Wang X.S., Booth J., Stuart J., McGregor I.S., Arnold J.C. (2016). ABC transporters P-gp and Bcrp do not limit the brain uptake of the novel antipsychotic and anticonvulsant drug cannabidiol in mice. PeerJ.

[79] Libro R., Scionti D., Diomede F., Marchisio M., Grassi G., Pollastro F., Piattelli A., Bramanti P., Mazzon E., Trubiani O. (2016). Cannabidiol modulates the immunophenotype and inhibits the activation of the inflammasome in human gingival mesenchymal stem cells. Front Physiol..

[80] Misri S., Kaul K., Mishra S., Charan M., Verma A.K., Barr M.P., Ahirwar D.K., Ganju R.K. (2022). Cannabidiol inhibits tumorigenesis in cisplatin-resistant non-small cell lung cancer via TRPV2. Cancers.

[81] dos-Santos-Pereira M., Guimaraes F.S., Del-Bel E., Raisman-Vozari R., Michel P.P. (2020). Cannabidiol prevents LPS-induced microglial inflammation by inhibiting ROS/NF-κB-dependent signaling and glucose consumption. Glia.

[82] Shrivastava A., Kuzontkoski P.M., Groopman J.E., Prasad A. (2011). Cannabidiol induces programmed cell death in breast cancer cells by coordinating the cross-talk between apoptosis and autophagy. Mol. Cancer Ther..

[83] Murase R., Kawamura R., Singer E., Pakdel A., Sarma P., Judkins J., Elwakeel E., Dayal S., Martinez-Martinez E., Amere M. (2014). Targeting multiple cannabinoid anti-tumour pathways with a resorcinol derivative leads to inhibition of advanced stages of breast cancer. Br. J. Pharmacol..

[84] Desprez P.-Y., Murase R., Limbad C., Woo R.W., Adrados I., Weitenthaler K., Soroceanu L., Salomonis N., McAllister S.D. (2021). Cannabidiol treatment results in a common gene expression response across aggressive cancer cells from various origins. Cannabis Cannabinoid Res..

[85] Bellio C., DiGloria C., Foster R., James K., Konstantinopoulos P.A., Growdon W.B., Rueda B.R. (2019). PARP inhibition induces enrichment of DNA repair–proficient CD133 and CD117 positive ovarian cancer stem cells. Mol. Cancer Res..

[86] Thakur B., Ray P. (2017). Cisplatin triggers cancer stem cell enrichment in platinum-resistant cells through NF-κB-TNFα-PIK3CA loop. J. Exp. Clin. Cancer Res..

[87] Namdar D., Anis O., Poulin P., Koltai H. (2020). Chronological review and rational and future prospects of cannabis-based drug development. Molecules.

[88] Mechoulam R., Ben-Shabat S. (1999). From gan-zi-gun-nu to anandamide and 2-arachidonoylglycerol: The ongoing story of cannabis. Nat. Prod. Rep..

[89] Russo E.B. (2019). The case for the entourage effect and conventional breeding of clinical cannabis: No “strain,” no gain. Front. Plant Sci..

[90] Guo X., Wang X.-F. (2009). Signaling cross-talk between TGF-β/BMP and other pathways. Cell Res..

